# Surgical therapy of primary hepatic angiosarcoma

**DOI:** 10.1186/s12893-018-0465-5

**Published:** 2019-01-10

**Authors:** Verena Tripke, Stefan Heinrich, Tobias Huber, Jens Mittler, Maria Hoppe-Lotichius, Beate K. Straub, Hauke Lang

**Affiliations:** 1 Department of General, Visceral and Transplantation Surgery, University Hospital of Mainz, Langenbeckstrasse 1, 55131 Mainz, Germany; 2 Institute of Pathology, University Hospital of Mainz, Mainz, Germany

**Keywords:** Liver resection, Surgical oncology, Liver malignancies

## Abstract

**Background:**

Primary hepatic angiosarcoma (PHA) is a rare tumor entity. Radical surgical resection is currently considered the best treatment choice. The aim of this analysis is to report our experience with surgery for PHA.

**Methods:**

All resections of PHA from 01/2002 until 06/2017 were identified from our prospective institutional database. All cases were re-confirmed by a second pathologist. We analyzed completeness of resection, overall (OS) and disease-free survival (DFS).

**Results:**

Nine patients with PHA underwent hepatic resection. Median follow-up after surgery was 15.5 months (range: 3–144). At last follow-up 4/9 patients were alive, three of them without recurrence 15, 21 and 144 months after surgery. Five patients developed PHA recurrence. Four of these died 3 to 17 months after surgery. One patient with PHA recurrence is alive 15 months after surgery. Another patient without PHA recurrence died 59 months after surgery from pancreatic cancer. Median OS and DFS after resection was 18 months (range: 3–144 months) and 10 months (range: 2–144 months), respectively. After R-0 resection (*n* = 8), the median OS and DFS was 59 and 11 months.

**Conclusions:**

Resection of PHA is the only approach to achieve complete tumor removal and offers a chance for long-term survival and should be evaluated in cases of PHA.

## Background

Primary hepatic angiosarcoma (PHA) is rare malignant tumor of endothelial origin, representing only 1–2% of all primary liver malignancies [1]. Accepted risk factors are the exposure to vinyl chloride monomers [2], arsenic salts [3] and thorotrast [4] as well as the use of androgenic steroids [5]. Patients usually present with unspecific symptoms such as abdominal pain, fever and weight loss. In almost 25% of cases the tumor is diagnosed after rupture and abdominal hemorrhage [6]. The majority of patients presents with large, often multifocal or even metastatic disease at the time of diagnosis [7].

Due to the rarity of the tumor there is hardly any data regarding best treatment and prognostic factors. Current treatment approaches are based on case reports and very small case series, only. Radical surgical resection seems to be the only curative treatment option for PHA [6]. Chemotherapy or transcatheter arterial chemoembolization (TACE) are used with palliative intention only in a palliative setting while transcatheter arterial embolization (TAE) has been reported to be effective in controlling acute bleeding [8, 9]. A multicenter retrospective study from Miller et al. suggests beneficial effects of transarterial radioembolization (TARE) for the palliative treatment of soft tissue sarcomas of the liver [10].

The aim of this study was to analyze our experience with hepatic resection for PHA and to compile data about surgical treatment in this rare malignancy.

## Methods

All patients who underwent surgery for PHA from 01/2002 until 06/2017 were identified from our prospective institutional liver surgery database. Data were analyzed with regard to patients` characteristics, clinical and perioperative parameters, completeness of resection as well as overall (OS) and disease-free survival (DFS). All cases were histologically examined and confirmed by a second pathologist. A preoperative biopsy positive for PHA was only available in three cases. Postoperative surgical complications were graded according to the Dindo-Clavien classification [11]. Follow-up ended on March 31, 2018.

### Statistics

Overall (OS) and disease-free survivals (DFS) were calculated from the date of surgery until death or tumor recurrence using the Kaplan-Meier method. Continuous variables were presented as median/range or mean/SD, whereas categorical variables were presented as numbers and percentages. SPSS Version 23 (IBM Corporation, USA) was used for statistical analysis.

## Results

Out of 2520 liver resections during the period of analysis, a total of 9 (0.4%) resections for PHA were identified. Patient’s characteristics are presented in Table 1.

**Table 1:** Patients` characteristics and operative data

Pat.	Age (yrs.)	Gender	Symptoms	ASA	Type of Surgery	Multi-focal	Tumor size (cm)	Radicality (R0/R1)/Grading	Complications	DFS (months)	OS (months)
1	62	Male	Abdominal pain	3	Segmentectomy	No	1.5	R0; G1	Postoperative hemorrhage (IIIb)	144	144 (alive)
2	48	Male	Abdominal pain	3	Bisegmentectomy	Yes	7.5	R0; G2	Postoperative hemorrhage (IIIb)	3.5	17 (deceased)
3	61	Female	Abdominal pain	2	Right hemihepatectomy	Yes	18	R0; G3	Gastrointestinal bleeding (II)	2.5	3 (deceased)
4	49	Male	Incidental finding on imaging	2	Left hemihepatectomy	Yes	4	R0; G2	Wound infection (I)	59	59 (deceased)
5	66	Male	Incidental finding on imaging	3	non-anatomical resection	No	1.5	R0; G2	Hepatic dysfunction (I)	8	11 (deceased)
6	74	Female	Abdominal pain	3	Bisegmentectomy	No	4	R1; G2	None	2.5	3 (deceased)
7	75	Male	Incidental finding during surgery	3	non-anatomical resection	No	1.3	R0; G1	None	21	21 (alive)
8	62	Female	Incidental finding during surgery	2	non-anatomical resection	No	1.8	R0; G1	None	15	15 (alive)
9	55	Female	Abdominal pain	3	Right hemihepatectomy	Yes	17	R0; G2–3	Bile leakage (IIIa)	7	14.5 (alive)

In 5/9 patients, tumors were symptomatic (abdominal pain) while in 4 cases PHA was diagnosed incidentally upon CT/MRI (*n* = 2) or during surgery for other indications (*n* = 2).

Three patients suffered from liver cirrhosis (Child A). There were 5 solitary and 4 multifocal intrahepatic PHA. No patient had extrahepatic metastatic disease. Preoperative biopsy was available in three cases. All these cases were positive for PHA, two were graded G1, while one case was graded G2. In one patient with multifocal PHA 6 cycles of neoadjuvant chemotherapy with paclitaxel were administered prior to resection. Operative procedures included major hepatectomy (*n* = 3), bisegmentectomy (*n* = 2) and segmentectomies or non-anatomical resections (*n* = 4). A complete resection (R0) was achieved in 8/9 of the cases. One resection of a multifocal PHA, that was intended to be a complete resection, was classified as R1 in the final histological report.

One patient with multifocal PHA received 5 cycles adjuvant therapy with doxorubicin and ifosfamid after surgery.

### Morbidity and mortality

Postoperative complications occurred in 6/9 patients. There were two grade I, one grade II, one grade IIIa (bile leakage requiring percutaneous drainage) and two grade IIIb (postoperative hemorrhages) complications, but no grade IV or V complication. Both 30- and 90-day postoperative mortality were 0%.

### Follow-up

The median follow-up was 15.5 months (range: 3–144 months).

At last follow-up 4 out of 9 patients were alive with 3 of them without recurrence 15, 21 and 144 months after surgery, respectively. In these patients, tumor grading of PHA was G1 each. One patient without tumor-recurrence died 59 months after surgery for PHA due to pancreatic cancer. He had received neoadjuvant chemotherapy with paclitaxel prior to resection. The histological grading was G2.

### Treatment of recurrence

Five patients developed tumor recurrence within 8 months after surgery (median 3.5 months). The histological grading of PHA had been G2 or G3.

Initial site of tumor recurrence was diffuse intrahepatic in all cases. Two of these five patients had initially multifocal disease and developed early recurrence within three months. Due to a poor general condition, these patients were unable to receive palliative chemotherapy. They died only two weeks after diagnosis of tumor recurrence. Two other patients received palliative chemotherapy with paclitaxel for tumor recurrence: one patient died 3 months after diagnosis of intrahepatic tumor recurrence (11 months after surgery), while the other patient was still alive at last follow-up for 14.5 months after surgery (and 7 months after diagnosis of recurrence). One patient, who developed intrahepatic tumor recurrence 3.5 months after surgery, was initially treated with TACE. During the course of disease, bone metastasis was diagnosed and a palliative chemotherapy with ifosfamid/doxorubicin was initiated. The patient survived for 17 months after surgery.

### Survival

**Fig. 1:**
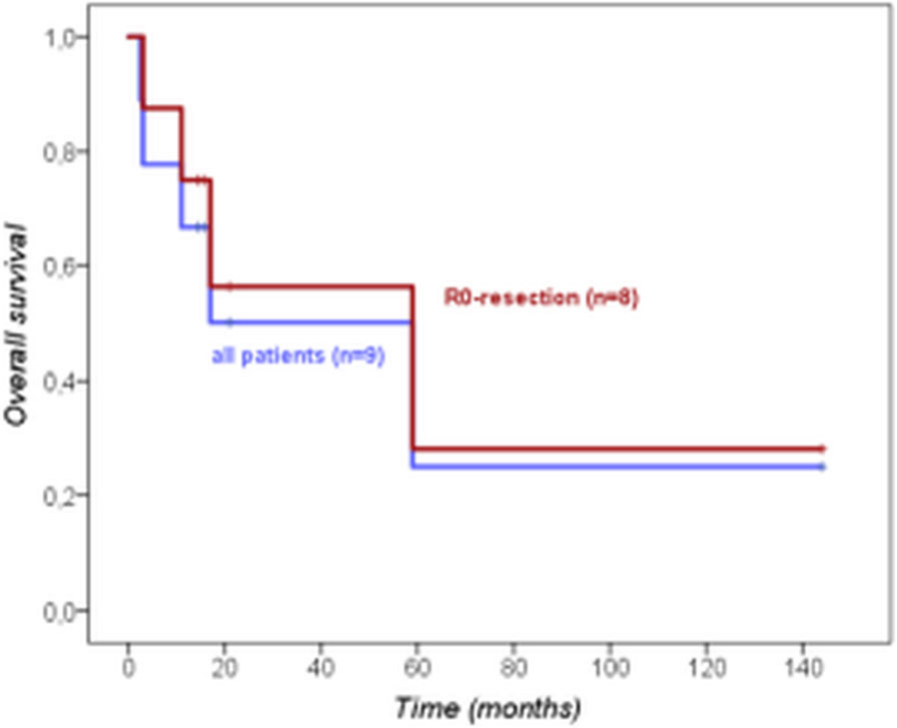
Overall survival of patients with PHA after surgery (*n* = 9) and after R0-resection (*n* = 8)

Median OS and DFS after resection was 18 months (range: 3–144 months) and 10 months (range: 2–144 months), respectively. After R0-resection (*n* = 8), the median OS and DFS was 59 and 11 months (Fig. 1).

## Discussion

Due to the low incidence of PHA the current literature is based on case reports and few very small case series, only. In the absence of established treatment guidelines radical surgical resection (R0 status) is currently considered the best treatment to give patients with PHA a decent chance for long-term survival [12–14].

Out of 2520 liver resections on a 15-year period, we identified 9 patients with histologically confirmed PHA. This is to our knowledge the largest case series of surgically treated patients with PHA from a single-center in the western world (Table 2).

**Table 2:** Case series of PHA

Author (year)	Country	Number	Operation	Other Treatment	Median survival
Molina et al. [1] (2003)	America	5	2 operation + chemotherapy, 1 operation	2 conservative	2 months
Weitz et al. [23] (2007)	America	5	3 operation (2 potentially curative)	2 no operation	n.a.^a^
Kim et al. [13] (2009)	Korea	5	–	4 chemotherapy, 1 conservative	3 months
Matthaei et al. [5] (2009)	Germany	5	5 operation	–	30 months
Park et al. [9] (2009)	Korea	6	–	4 TACE, 2 TAE	3.5 months
Zhou et al. [12] (2010)	China	6	1 operation, 5 operation + TACE	–	12 months
Huang et al. [24] (2011)	China	9	3 operation + chemotherapy, 1TAE + operation	4 conservative, 1 TAE + TACE	4 months
Chi et al. [25] (2011)	China	7	3 operation, 2 liver transplantation	2 conservative	6.5 months
Duan et al. [7] (2012)	China	6	5 operation, 1 operation + chemotherapy 1 operation + chemotherapy,	–	40.5 months
Bruegel et al. [21] (2012)	Germany	7	1 operation	4 chemotherapy	16 months
Hur et al. [26] (2015)	Korea	8	1 operation	1 TACE, 4 chemotherapy, 2 conservative	214 days
Huang et al. [14] (2016)	Taiwan	6	1 operation	1 chemotherapy, 1 chemotherapy + cyberknife, 1 cyberknife, 2 conservative	n.a.
Zhu et al. [27] (2015)	China	2	2 operation	–	12 months
Present study (2018)	Germany	9	9 operation	–	18 months

n.a. not available; ^a^both patients with potentially curative resection died within 11 months after surgery

**Fig. 2:**
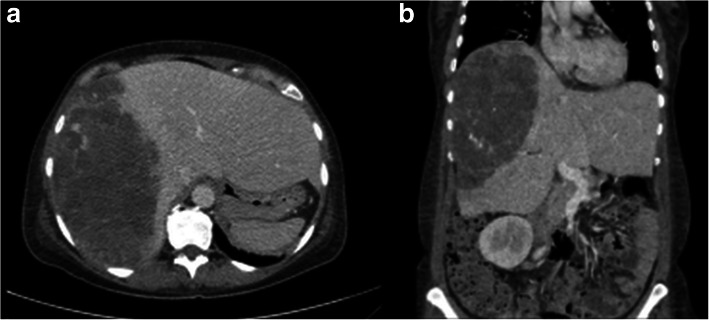
Preoperative contrast enhanced computed tomography scan showed a heterogenous mass in the right liver lobe (**a** and **b**)

In our series the median postoperative survival was 18 months in all patients and 59 months in case of R0-resection. A meta-analysis of 64 cases from Zheng et al. reports a median survival of 5 months for the entire collective of patients with PHA, whereas patients (*n* = 30 in total) having complete tumor resection alone or in combination with adjuvant chemotherapy had a median OS of 17 months [6]. Patients with a PHA confined to the liver had a longer median survival than those with metastatic tumors (9 months versus 3 months). Consistently, it is not surprising that patients with a small solitary PHA tend to have a better prognosis than those with large or multifocal tumors (Fig. 2). In our series, there were even 4 cases (out of 8 with R0-resection) where the PHA had been diagnosed incidentally. The detection and treatment in a very early stage of disease is the most obvious reason for the overall survival of even 59 months after R0-resection in our series which is remarkably better than all data reported before.

In our series patients with multifocal PHA had a high rate of very early intrahepatic recurrence, all finally resulting in liver failure within a few months. In some patients, general status deteriorated rapidly preventing even any palliative treatment. Nevertheless, surgical resection seems to be at least an option not only to remove tumor burden but also to offer the chance for long-term survival, as shown in one of our patients who survived more than 10 years without recurrence. Similarly, Matthaei et al. reported on one long-term tumor-free survival of more than 8 years in 1 of 5 patients after complete resection [15]. In view of the low perioperative morbidity, these data justify surgical resection as a radical approach for PHA, the more, as PHA is considered an absolute contraindication for liver transplantation, currently. There is only one 5 year-survivor after liver transplantation (LT) in the literature. In general, the outcome of LT for PHA is very poor [16–19]. In a report of 22 cases of LT for PHA, the median survival was 6 months. Five of the 22 patients died due to infections and 17 due to tumor recurrence [17].

Therefore, the herein presented data as well as the few data from literature suggest that the option of surgical resection should be evaluated in all patients with PHA confined to the liver. Depending on the tumor burden and considering patients` general condition as well as the patients` will, even extended surgery can be performed with very low mortality.

**Fig. 3:**
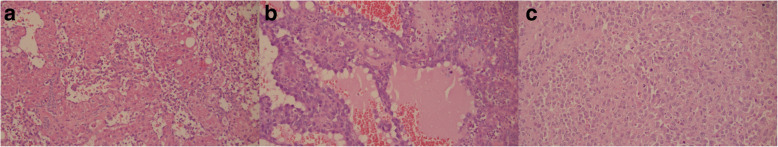
Pathology findings (hematoxylin and eosin staining, × 200) showing sinusoidal and spindle-shape growth of the malignant endothelial cells: G1 (**a**), G2 (**b**) and G3 (**c**)

Histological grading seems also to be important to predict patients` prognosis [20]. In our series, patients with tumor recurrence had mainly G2 or G3 tumors, whereas patients without tumor recurrence had well-differentiated (G1) or G2 tumors (Fig. 3). The value of preoperative biopsy is controversial as a representative probe cannot be guaranteed, if samples were taken from necrotic areas or surrounding liver parenchyma. Besides, percutaneous biopsy carries a considerable risk for intraabdominal bleeding and may induce metastatic spread.

In our series preoperative biopsy positive for PHA was available in three cases. Biopsy was graded G1 in one case. However, final histology after resection revealed a G3 tumor, so the biopsy had not been representative for the whole tumor. Therefore the value of preoperative biopsy as a prognostic tool is debatable.

Of note, patients with tumor rupture have a dismal prognosis of often less than one month [6, 9].

Preoperative imaging via computed tomographic scan or magnetic resonance is also a challenge as differentiation from other liver tumors like hemangioma, intrahepatic cholangiocarcinoma or metastases is difficult as PHA shares similar imaging characteristics [21].

Currently, there is no evidence for the efficacy of chemotherapy. Some very small case series suggest a survival benefit for patients who receive palliative chemotherapy with adriamycin/cisplatin/ifosamid/paclitaxel (two patients surviving for more than 14 months after diagnosis; [8]) or doxorubicin/carboplatin and 5-fluorouracil (2 patients surviving 9 and 16 months, respectively; [13]).

The Angiotax study of weekly paclitaxel for angiosarcoma reported median OS of 8 months [22]. Chemotherapy is considered a treatment option in cases of unresectable or metastatic disease. Currently, the value of neoadjuvant chemotherapy remains unclear.

In few papers, the use of transarterial embolization (TAE) or transarterial-chemoembolization (TACE) has been addressed. While TAE is effective in the management of acute bleeding due to rupture, TACE is mainly used in palliative oncological intention. The survival after TACE in few cases ranges up to one year [9].

## Conclusion

In conclusion, oncological treatment data on PHA is very limited at the moment as only few case reports and very small series exist in the literature. In the absence of effective chemotherapy or other tumor directed treatment modalities, and despite high recurrence rates, radical surgical resection is the best approach to achieve complete tumor removal and, although rarely, offer a chance for long-term survival in patients with PHA.

## Abbreviations

DFS: Disease-free survival
LT: Liver transplantation
OS: Overall survival
PHA: Primary hepatic angiosarcoma
TACE: Transcatheter arterial chemoembolization
TAE: Transcatheter arterial embolization
TARE: Transarterial radioembolization
